# Supplementary material for: Circadian and diel regulation of photosynthesis in the bryophyte Marchantia polymorpha
Source: Plant Cell Environ. 2022 Jun 3;45(8):2381–94. doi: 10.1111/pce.14364 (PMC9546472; doi:10.1111/pce.14364)
Supplement: Supplementary file 1 — Supporting information. [file PCE-45-2381-s002.pdf]

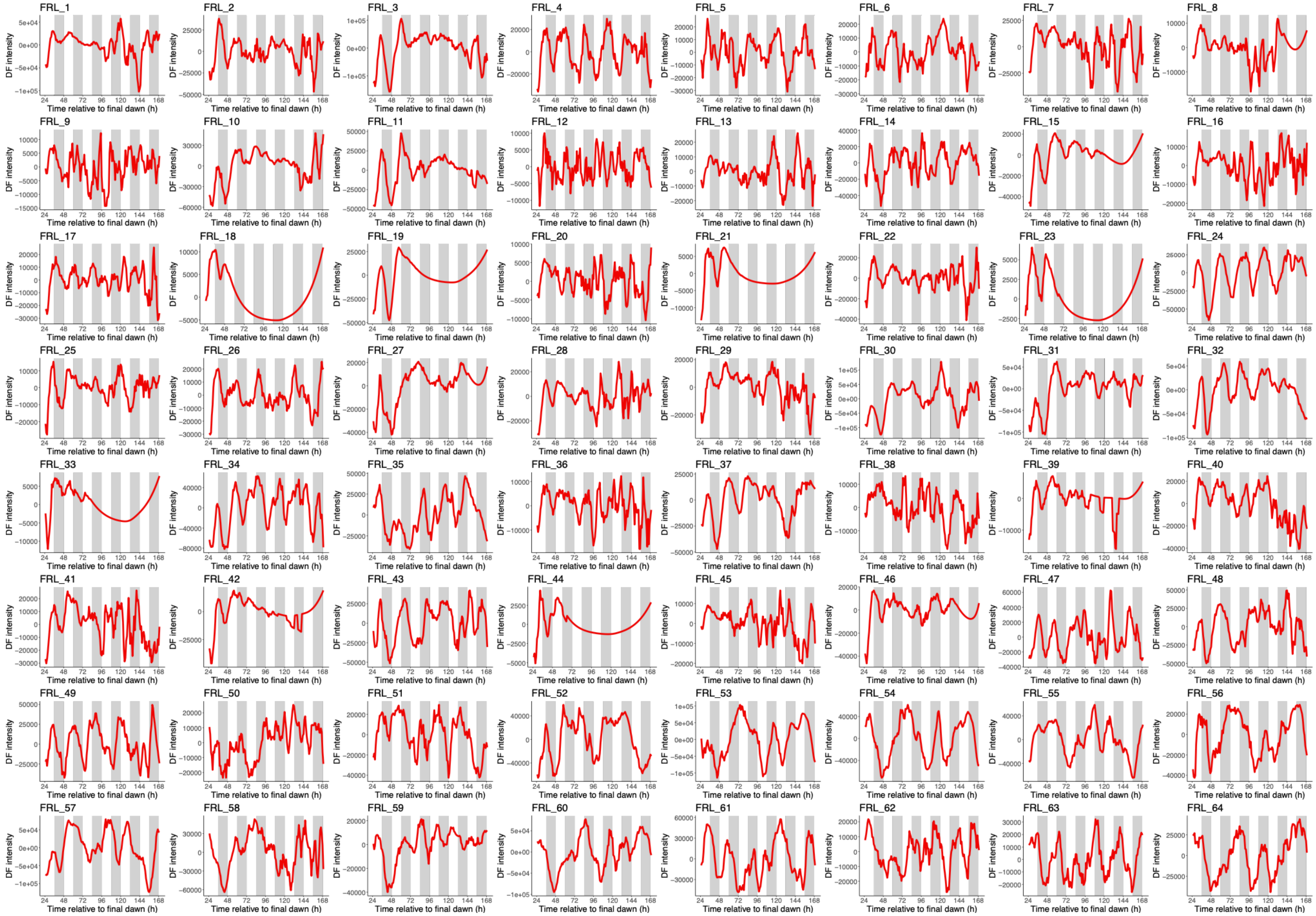
FRL_1
FRL_2
FRL_3
FRL_4
FRL_5
FRL_6
FRL_7
FRL_8
FRL_9
FRL_10
FRL_11
FRL_12
FRL_13
FRL_14
FRL_15
FRL_16
FRL_17
FRL_18
FRL_19
FRL_20
FRL_21
FRL_22
FRL_23
FRL_24
FRL_25
FRL_26
FRL_27
FRL_28
FRL_29
FRL_30
FRL_31
FRL_32
FRL_33
FRL_34
FRL_35
FRL_36
FRL_37
FRL_38
FRL_39
FRL_40
FRL_41
FRL_42
FRL_43
FRL_44
FRL_45
FRL_46
FRL_47
FRL_48
FRL_49
FRL_50
FRL_51
FRL_52
FRL_53
FRL_54
FRL_55
FRL_56
FRL_57
FRL_58
FRL_59
FRL_60
FRL_61
FRL_62
FRL_63
FRL_64
DF intensity
Time relative to final dawn (h)
24 48 72 96 120 144 168

Figure S1. Example of raw delayed fluorescence data for 64 individual replicate thalli of *M. polymorpha*, under free running light conditions. Clear areas and shaded areas on the plots indicate subjective day and night, respectively.
