# Supplementary material for: Circadian and diel regulation of photosynthesis in the bryophyte Marchantia polymorpha
Source: Plant Cell Environ. 2022 Jun 3;45(8):2381–94. doi: 10.1111/pce.14364 (PMC9546472; doi:10.1111/pce.14364)
Supplement: Supplementary file 2 — Supporting information. [file PCE-45-2381-s006.pdf]

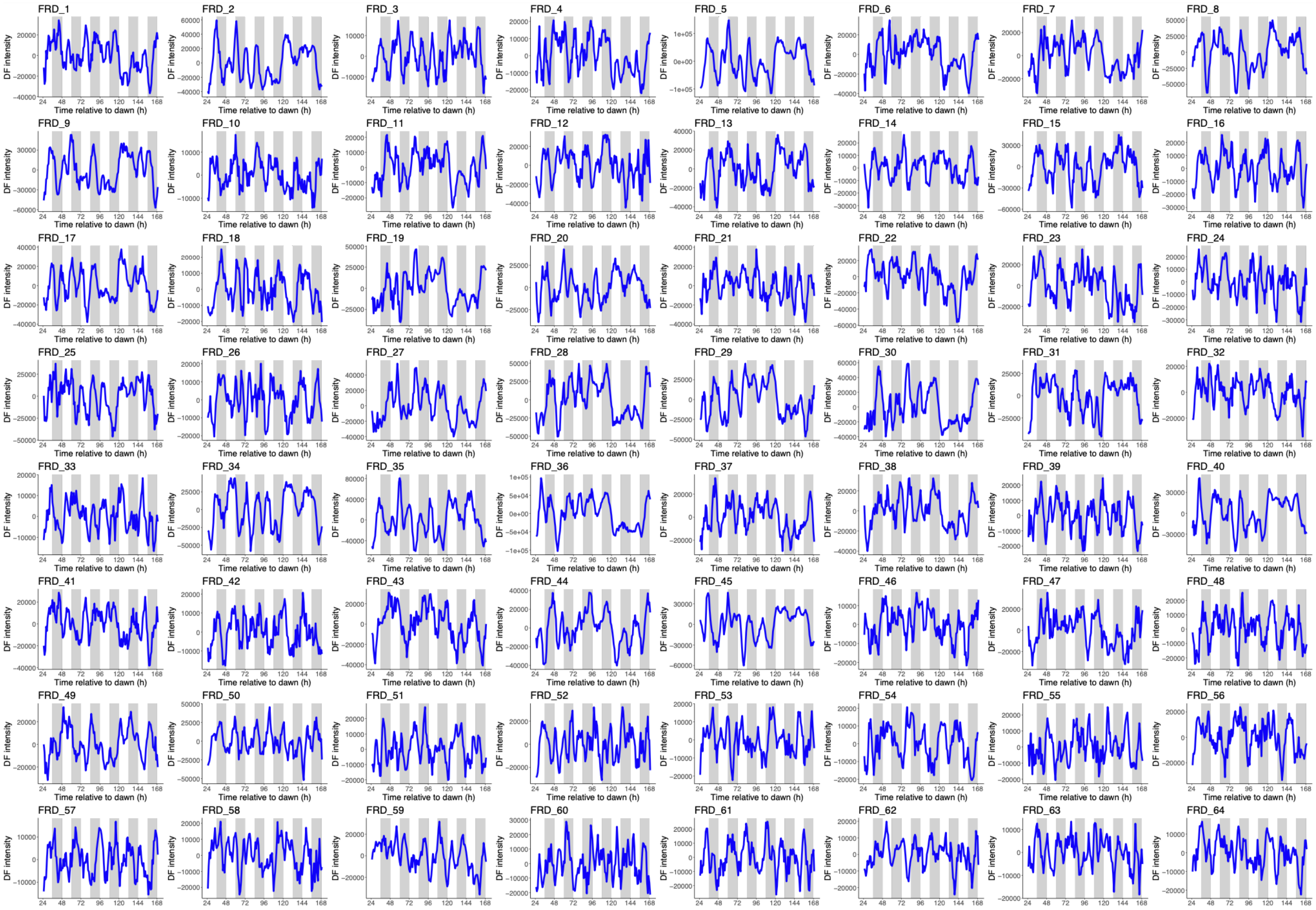
FRD_1
FRD_2
FRD_3
FRD_4
FRD_5
FRD_6
FRD_7
FRD_8
FRD_9
FRD_10
FRD_11
FRD_12
FRD_13
FRD_14
FRD_15
FRD_16
FRD_17
FRD_18
FRD_19
FRD_20
FRD_21
FRD_22
FRD_23
FRD_24
FRD_25
FRD_26
FRD_27
FRD_28
FRD_29
FRD_30
FRD_31
FRD_32
FRD_33
FRD_34
FRD_35
FRD_36
FRD_37
FRD_38
FRD_39
FRD_40
FRD_41
FRD_42
FRD_43
FRD_44
FRD_45
FRD_46
FRD_47
FRD_48
FRD_49
FRD_50
FRD_51
FRD_52
FRD_53
FRD_54
FRD_55
FRD_56
FRD_57
FRD_58
FRD_59
FRD_60
FRD_61
FRD_62
FRD_63
FRD_64
DF intensity
Time relative to dawn (h)
24 48 72 96 120 144 168

Figure S2. Example of raw delayed fluorescence data for 64 individual replicate thalli of *M. polymorpha*, under free running dark conditions. Clear areas and shaded areas on the plots indicate subjective day and night, respectively.
