# Supplementary material for: Circadian and diel regulation of photosynthesis in the bryophyte Marchantia polymorpha
Source: Plant Cell Environ. 2022 Jun 3;45(8):2381–94. doi: 10.1111/pce.14364 (PMC9546472; doi:10.1111/pce.14364)
Supplement: Supplementary file 3 — Supporting information. [file PCE-45-2381-s001.pdf]

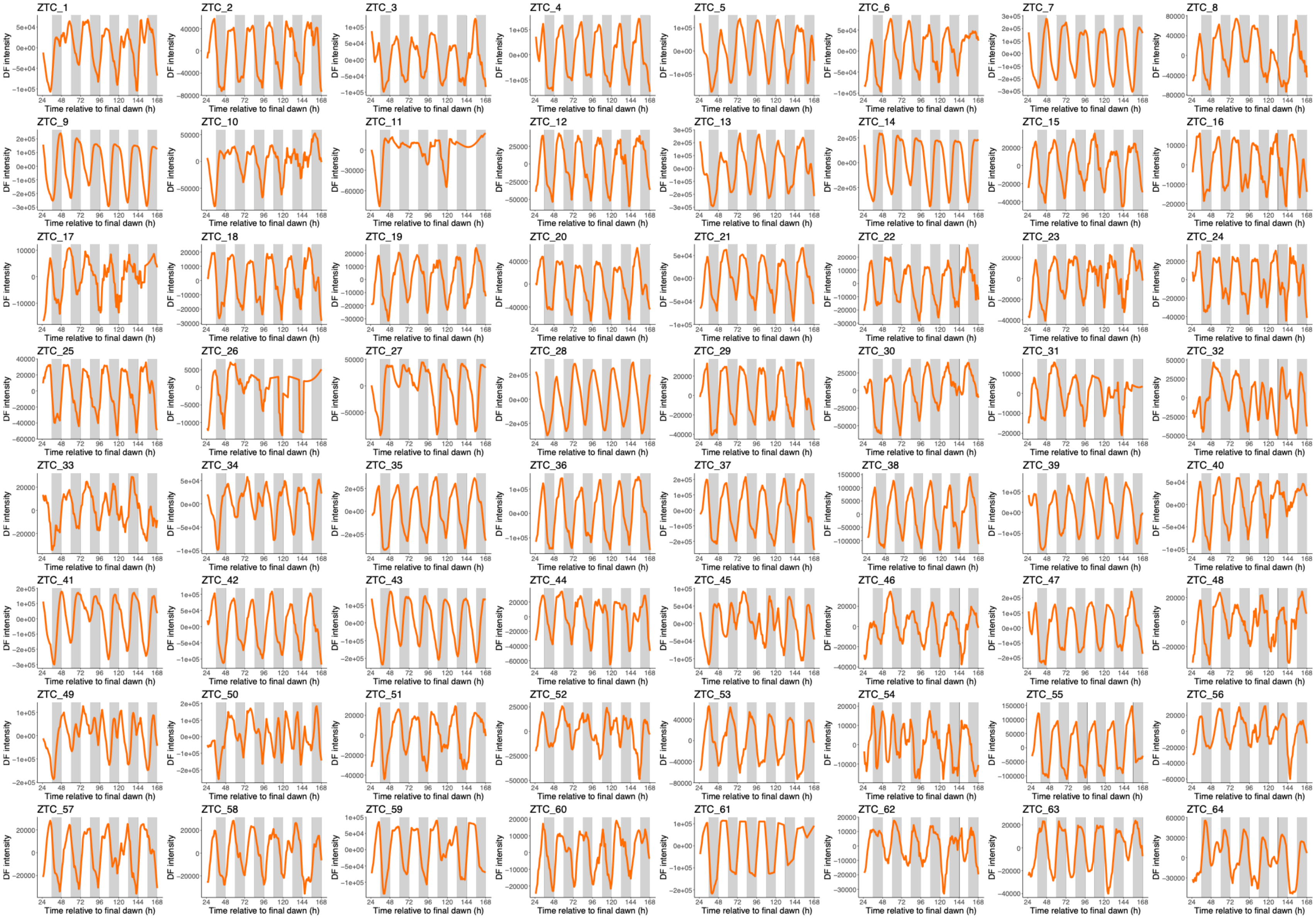
ZTC_1
ZTC_2
ZTC_3
ZTC_4
ZTC_5
ZTC_6
ZTC_7
ZTC_8
ZTC_9
ZTC_10
ZTC_11
ZTC_12
ZTC_13
ZTC_14
ZTC_15
ZTC_16
ZTC_17
ZTC_18
ZTC_19
ZTC_20
ZTC_21
ZTC_22
ZTC_23
ZTC_24
ZTC_25
ZTC_26
ZTC_27
ZTC_28
ZTC_29
ZTC_30
ZTC_31
ZTC_32
ZTC_33
ZTC_34
ZTC_35
ZTC_36
ZTC_37
ZTC_38
ZTC_39
ZTC_40
ZTC_41
ZTC_42
ZTC_43
ZTC_44
ZTC_45
ZTC_46
ZTC_47
ZTC_48
ZTC_49
ZTC_50
ZTC_51
ZTC_52
ZTC_53
ZTC_54
ZTC_55
ZTC_56
ZTC_57
ZTC_58
ZTC_59
ZTC_60
ZTC_61
ZTC_62
ZTC_63
ZTC_64
DF intensity
Time relative to final dawn (h)
24 48 72 96 120 144 168

Figure S3. Example of raw delayed fluorescence data for 64 individual replicate thalli of *M. polymorpha*, under zeitgeber cycles. Clear areas and shaded areas on the plots indicate day and night, respectively.
