# Supplementary material for: Circadian and diel regulation of photosynthesis in the bryophyte Marchantia polymorpha
Source: Plant Cell Environ. 2022 Jun 3;45(8):2381–94. doi: 10.1111/pce.14364 (PMC9546472; doi:10.1111/pce.14364)
Supplement: Supplementary file 4 — Supporting information. [file PCE-45-2381-s004.pdf]

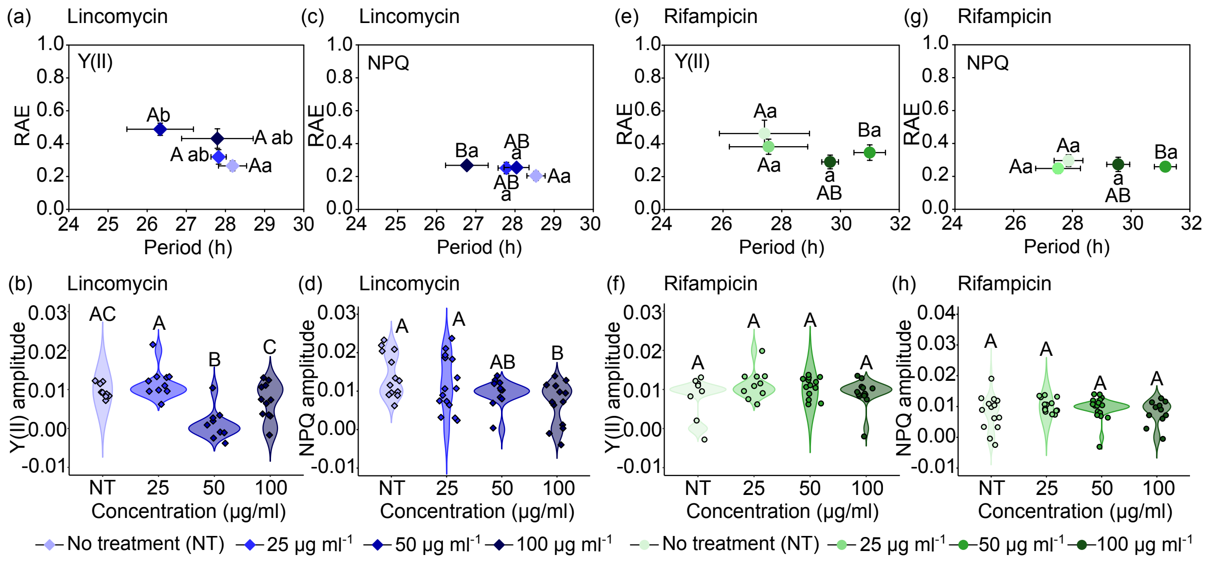

Figure S4. Amplitude of circadian rhythm of chlorophyll fluorescence parameters is reduced by the inhibitor of chloroplast translation lincomycin. This is a second direct repeat of the experiment in Fig. 4. (a-d) Effect of range of concentrations of lincomycin upon the circadian oscillation of (a, b) the apparent quantum yield of PSII (Y(II)) and (c, d) non-photochemical quenching of chlorophyll fluorescence. (e-h) Effect of range of concentrations of rifampicin upon the circadian oscillation of (e, f) Y(II) and (g, h) non-photochemical quenching of chlorophyll fluorescence. (a, c, e, g) Different letters next to data points indicate significant differences in period (upper case) and RAE (lower case), with data plotted ± s.e.m. (b, d, f, h) Different letters indicate significantly different mean phases. Analyzed with one-way ANOVA, where $p < 0.05$ based on Tukey's post-hoc test; n = 25 thalli.
