# Supplementary material for: Circadian and diel regulation of photosynthesis in the bryophyte Marchantia polymorpha
Source: Plant Cell Environ. 2022 Jun 3;45(8):2381–94. doi: 10.1111/pce.14364 (PMC9546472; doi:10.1111/pce.14364)
Supplement: Supplementary file 5 — Supporting information. [file PCE-45-2381-s003.pdf]

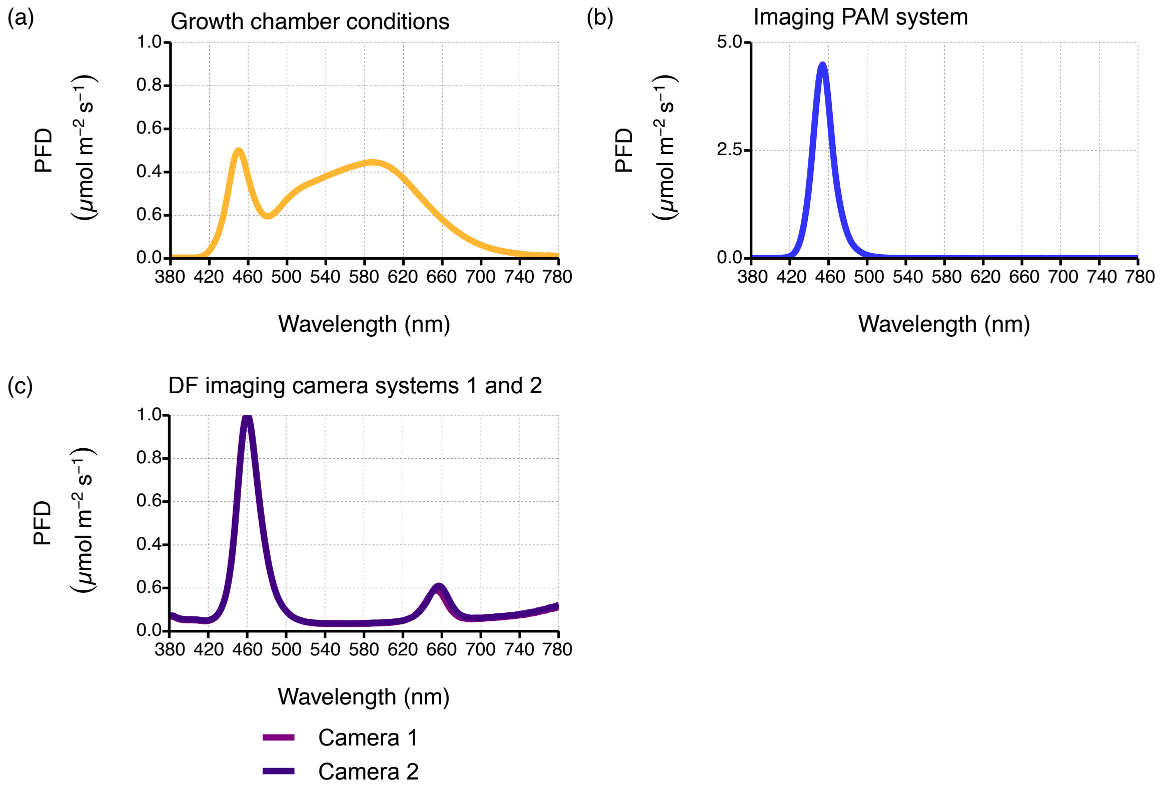

Figure S5. Spectra of light conditions used for experiments. Photon flux density spectrum within (a) growth chambers used for cultivation and entrainment of M. polymorpha, (b) imaging PAM system actinic light conditions and (c) delayed fluorescence imaging LED array.
