# Supplementary material for: Circadian and diel regulation of photosynthesis in the bryophyte Marchantia polymorpha
Source: Plant Cell Environ. 2022 Jun 3;45(8):2381–94. doi: 10.1111/pce.14364 (PMC9546472; doi:10.1111/pce.14364)
Supplement: Supplementary file 6 — Supporting information. [file PCE-45-2381-s007.pdf]

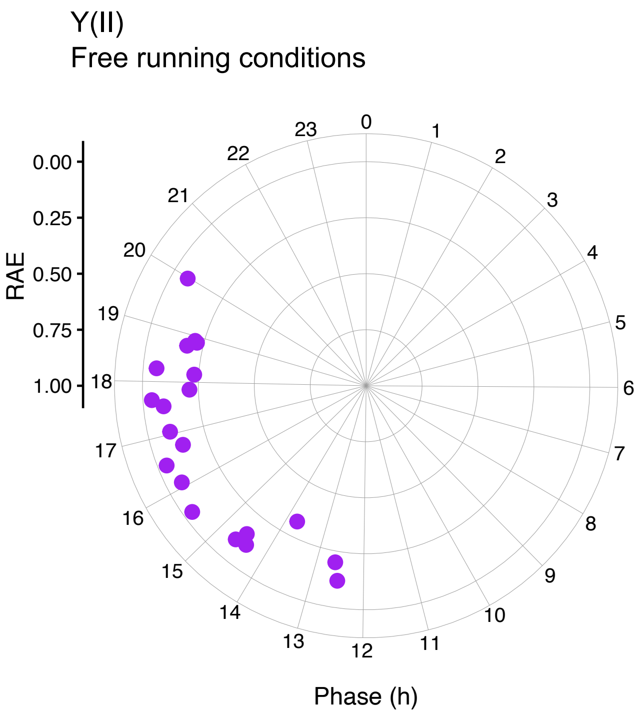

Figure S6. Phase distribution for Y(II) in *M. polymorpha* under free running conditions measured using PAM fluorescence. Phases of replicate thalli relative to dawn (0), calculated using fast Fourier transform-non linear least squares method (FFT-NLLS). Radial scale shows relative amplitude error (RAE) of each thallus, with RAE=0 at the exterior and RAE=1 at the centre of the circles, respectively.
